# Cannabis Consumption Systemic Adverse Effects

**DOI:** 10.5812/ijhrba.18627

**Published:** 2014-05-28

**Authors:** Maurizio Coppola, Raffaella Mondola

**Affiliations:** 1 Department of Addiction, Asl CN2, 12051, Alba (CN), Italy; 2 Department of Mental Health, ASL CN1, 12037, Saluzzo (CN), Italy

**Keywords:** Cannabis, Adverse Effects, Substance-Related Disorders

Dear Editor,

In the article entitled "Laboratory profiles of treatment-seeking subjects with concurrent dependence on cannabis and other substances: A comparative study", authors have focused on the systemic adverse effects of cannabis consumption through the evaluation of routine hematological parameters. Cannabis is extensively studied for its effects on the central nervous system, while its long term effects on other organs and systems are not much considered. Endocannabinoid system plays a key role in the modulation of hypothalamic-pituitary-adrenal axis and sympathetic nervous system. It is involved in regulating various cardiovascular activities such as heart rate, blood pressure and the baroreflex. Data emerged from studies performed on patients affected by myocardial infarction have shown that cannabis consumption increases the risk of myocardial infarction in the hour after use, as well as the mortality in a dose-dependent manner (1). Moreover, a growing body of evidences suggests that CB1 receptors exert influence over the development of insulin resistance and liver lipogenesis. On the other hand, CB2 receptors can cause liver inflammation and antifribogenic activity. Overall, clinical and experimental studies have confirmed the involvement of endocannabinoids in the pathogenesis of liver steatosis and cirrhosis. Data emerged from the studies performed on patients affected by hepatitis C have indicated the association between severe steatosis and cannabis consumption (2). Additionally, endocannabinoid receptors, in particular the CB2 type, are involved in the development and modulation of immune and hematologic cells. Information derived from studies performed on chronic cannabis consumers have shown that this substance can reduce the number of T and B lymphocytes and increase the number of eosinophils (3). Although the cannabis consumption is not associated with severe acute effects on health, clinical information have evidenced that its chronic use can produce significant alterations in many biological systems. Thus, patients should be discouraged from the recreational use of cannabis. Furthermore, the misleading message that cannabis is a soft and low risk drug should be reconsidered, given the growing scientific information demonstrating the contrary. Quraishi et al. promoted a more complete and adequate medical assessment of cannabis dependent patients in the absence of sophisticated diagnostic instruments. A better international cooperation is of great importance in order to promote common guidelines for monitoring and preventing the adverse effects of cannabis consumption.

## References

[1] Hall W, Degenhardt L (2009). Adverse health effects of non-medical cannabis use.. Lancet..

[2] Alswat KA (2013). The role of endocannabinoids system in fatty liver disease and therapeutic potentials.. Saudi J Gastroenterol..

[3] Yahya MD, Watson RR (1987). Immunomodulation by morphine and marijuana.. Life Sci..

